# CCR5 as a prognostic biomarker correlated with immune infiltrates in head and neck squamous cell carcinoma by bioinformatic study

**DOI:** 10.1186/s41065-022-00251-y

**Published:** 2022-09-27

**Authors:** Chunhong Li, Shanlin Chen, Chuanyu Liu, Chune Mo, Weiwei Gong, Jiahua Hu, Min He, Lei Xie, Xianliang Hou, Jianhong Tang, Minglin Ou

**Affiliations:** 1 Central Laboratory, Guangxi Health Commission Key Laboratory of Glucose and Lipid Metabolism Disorders, The Second Affiliated Hospital of Guilin Medical University, Guilin, Guangxi 541199 PR China; 2 Department of Pharmacy, Guangxi Health Commission Key Laboratory of Glucose and Lipid Metabolism Disorders, The Second Affiliated Hospital of Guilin Medical University, Guilin, Guangxi 541199 PR China; 3 College of Pharmacy, Guilin Medical University, Guilin, Guangxi 541199 PR China; 4 Department of Pharmacy, The Affiliated Hospital of Guilin Medical University, Guilin, Guangxi 541001 PR China

**Keywords:** Head and neck squamous cell carcinoma, C-C chemokine receptor 5, Tumor-infiltrating lymphocytes, Prognostic biomarker, Immune infiltration

## Abstract

**Background:**

C-C chemokine receptor 5 (CCR5) has recently been recognized as an underlying therapeutic target for various malignancies. However, the association of CCR5 with prognosis in the head and neck squamous cell carcinoma (HNSC) patients and tumor-infiltrating lymphocytes (TILs) is unclear.

**Methods:**

In the current experiment, methods such as the Tumor Immune Estimation Resource Analysis (TIMER), Gene Expression Profiling Interactive Analysis (GEPIA), UALCAN, and Kaplan-Meier plotter Analysis were used to comprehensively evaluate the expression of CCR5 in human various malignancies and the clinical prognosis in HNSC patients. Subsequently, we used the TIMER database and the TISIDB platform to investigate the correlation between CCR5 expression levels and immune cell infiltration in the HNSC tumor microenvironment. Furthermore, immunomodulatory and chemokine profiling were performed using the TISIDB platform to analyse the correlation between CCR5 expression levels and immunomodulation in HNSC patients.

**Results:**

We found that CCR5 expression in HNSC tumor tissues was significantly upregulated than in normal tissues. In HNSC, patients with high CCR5 expression levels had worse overall survival (OS, HR = 0.59, *p* = 0.00015) and worse recurrence-free survival (RFS, HR = 3.27, *p* = 0.00098). Upregulation of CCR5 expression is closely associated with immunomodulators, chemokines, and infiltrating levels of CD4+ T cells, neutrophils, macrophages, and myeloid dendritic cells. Furthermore, upregulated CCR5 was significantly associated with different immune markers in the immune cell subsets of HNSC.

**Conclusions:**

High expression of CCR5 plays an important prognostic role in HNSC patients and may serve as a prognostic biomarker correlated with immune infiltration, and further studies are still needed to investigate therapeutic targeting HNSC patients in the future.

## Introduction

Head and neck squamous cell carcinoma (HNSC) are the sixth most common cancer in the world. It is highly aggressive and locally progressive, with lymph node metastases, and approximately 900,000 new patients are diagnosed each year [1–3]. It originates from the mucosal epithelium of the lips, mouth, nasal passages, sinuses, nasopharynx, mid-pharynx, larynx, and hypopharynx, and is mainly correlated with smoking, hard-drinking, and the human papillomavirus infection (HPV). Most patients with HNSCs are treated with surgery, standard cytotoxic chemotherapy, radiation therapy, and anticancer drugs, but the overall response remains insufficient, and local recurrence or distant metastasis is common [4, 5]. China is undergoing rapid social and economic changes that may affect the incidence of cancer, especially lifestyle-related cancers such as HNSC. Its incidence has been on the rise in recent years. Furthermore, patients with recurrent or metastatic HNSC (R/M) have a poor prognosis. Immunotherapy is currently an effective treatment option for a variety of malignancies, including HNSC patients with R/M disease [6]. However, in the absence of specific immune-related biomarkers, the five -year survival rates of HNSC patients remains low.

As a seven-transmembrane G protein-coupled receptor (GPCR), CC chemokine receptor 5 (CCR5) can regulate the transport and the effector functions of a variety of immune cells [7]. CCR5 promotes angiogenesis by promoting endothelial cell migration, proliferation, angiogenesis and VEGF secretion. In situ expression of the CCR5 can activate the Ca^2+^ signaling pathway, which facilitates the differentiation and metastasis of the regulatory T cells (Tregs) to the part of inflammation, and is involved in stimulating tumor cell proliferation, infiltration, and migration through various mechanisms [8, 9]. Besides being a co-receptor of HIV, CCR5 is closely related to breast cancer, colon cancer, pancreatic cancer, glioblastoma multiforme, liver cancer and other cancers, and has been identified as a potential therapeutic target for various malignant tumors [10–14]. However, the functional role and immunomodulatory mechanisms of CCR5 in HNSC are unclear.

In this study, methods such as the TIMER database analysis, GEPIA platform analysis, UALCAN database analysis, and Kaplan-Meier plotter analysis were used to comprehensively evaluate the expression levels of CCR5 in HNSC patients and its correlation with prognosis. Subsequently, we researched the relevance of CCR5 expression levels and the immune infiltration in HNSC tumor microenvironment using the TIMER database and the TISIDB platform. Furthermore, immunomodulatory and chemokine profiling was performed using the TISIDB platform resources to analyse the relevance between CCR5 expression levels and immunomodulation for HNSC patients. Our findings suggested that the CCR5 is highly expressed in in HNSC patients, and possible links and mechanisms of CCR5 regulation in TILs.

## Materials and methods

### CCR5 expression analysis in various human malignancies

The expression levels of CCR5 in the various malignancies were analyzed using the TIMER 2.0 (http://timer.cistrome.org/), which is web server provides comprehensive analysis and visualization functions of tumor infiltrating immune cells [15]. Among them, 520 were HNSC patients and 44 were healthy controls in the TIMER 2.0 database. The expression data of CCR5 in the tumors without normal tissue samples in the Cancer Genome Atlas (TCGA) database and the reciprocal normal specimens of the Genotype-Tissue Expression database (GTEx) were acquired and analyzed using the GEPIA analysis (http://gepia2.cancer-pku.cn/#index), which is an enhanced web server for large-scale expression profiling and interactive analysis [16]. To further validate the expression levels of CCR5 in different cancer types, we performed the UALCAN database analysis (http://ualcan.path.uab.edu/), which is a web portal for facilitating tumor subgroup gene expression and survival analyses [17] on different tumor types and the reciprocal normal specimens.

### Clinical-pathological parameters analysis

Kaplan-Meier plotter analysis (https://kmplot.com/analysis/) that is web-based survival analysis tool tailored for medical research was adopted to analyze the relevance between CCR5 expression levels and the survival situation in HNSC patients [18]. Specifically, the relevance between the expression levels of CCR5 and clinical prognostic significance in HNSC patients was explored using the UALCAN and Kaplan-Meier plots in key clinicopathological parameters (e.g., patient’s gender, patient’s races, cancer stages, cancer grades, and lymph node stage). We employed the Kaplan-Meier plots to analyze the clinical prognostic significance of CCR5 in HNSC patients, including overall survival (OS) profiling and recurrence-free survival (RFS) profiling. Hazard ratios (HR) with 95% confidence intervals were estimated, along with log-rank *p*-values. *p*-values < 0.05 was defined as significant difference.

### Immune cell infiltration analysis

To investigate the relevance between the expression levels of CCR5 and immune infiltration in HNSC patients, the immune cell infiltration profiling was explored using the TIMER and TISIDB platform resources (http://cis.hku.hk/TISIDB/index.php), which is an integrated repository portal for tumor–immune system interactions [19]. Specifically, we examined the relevance between CCR5 expression levels and TILs levels, including CD8+ T cells, B cells, monocytes, TAMs, M1 macrophages, M2 macrophages, neutrophils, and dendritic cells. According to the relevant control modules, the relevance between CCR5 expression levels with immunogenic markers of TILs has been further investigated, including immunological biomarkers of CD8+/CD4+ T cells, B cells, monocytes, natural killer cells, dendritic cells, TAMs, M1 macrophages, M2 macrophages, neutrophils, T cells, and other related cell subtypes.

### Immunomodulators and chemokine analysis

To further explore the relevance between CCR5 expression levels and immune regulation in HNSC patients, immunomodulators and chemokine analysis was performed using the TISIDB platform. Specifically, we investigated the relevance of CCR5 expression with 45 immunostimulators, 24 immunoinhibitors, 41 chemokines, and 18 receptors in HNSC patients.

### Statistical analysis

Kaplan-Meier plots were used to structure survival curves. For the Kaplan-Meier, GEPIA and TISIDB plots, HR and *p*-values were detected by log-rank test. Spearman correlation coefficients were analyzed to explore the relationship between CCR5 expression levels and immune cell infiltration, immunomodulators and chemokine levels. Relevance strength was assessed as follows: < 0.2 for a very weak relevance, < 0.4 for a weak relevance, < 0.6 for a moderate relevance, < 0.8 for a strong relevance, and < 1.0 for a very strong relevance. p-values < 0.05 was defined as significant difference.

## Results

### High CCR5 expression levels in head and neck squamous cell carcinoma

**Fig. 1:**
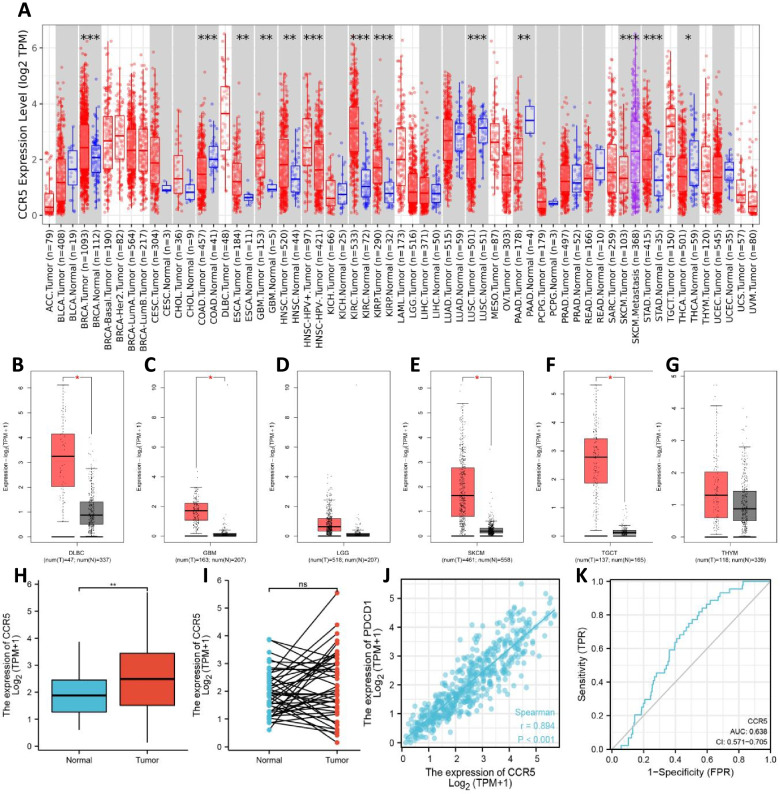
The expression levels of CCR5 in the various malignancies. **A** Upregulated or downregulated CCR5 in human various malignancies from TCGA database were determined by TIMER 2.0 (*p < 0.05, **p < 0.01, ***p < 0.001). **B-G** For the type of DLBC, GBM, LGG, SKCM, TGCT, and THYM in the TCGA project, the corresponding normal tissues of the GTEx database were included as controls. The box plot data were supplied (*p < 0.05, **p < 0.01, ***p < 0.001). (H). Upregulated CCR5 expression in HNSC tissue specimens compared with the normal tissue specimens from TCGA database. **I** The mRNA level of CCR5 in pairs of HNSC tissue specimens and their paired normal adjacent tissue specimens. **J** The correlation analysis between CCR5 and PD1 (PDCD1) mRNA level. **K** The receiver-operating characteristic (ROC) curve analysis of CCR5 in HNSC patients

**Fig. 2:**
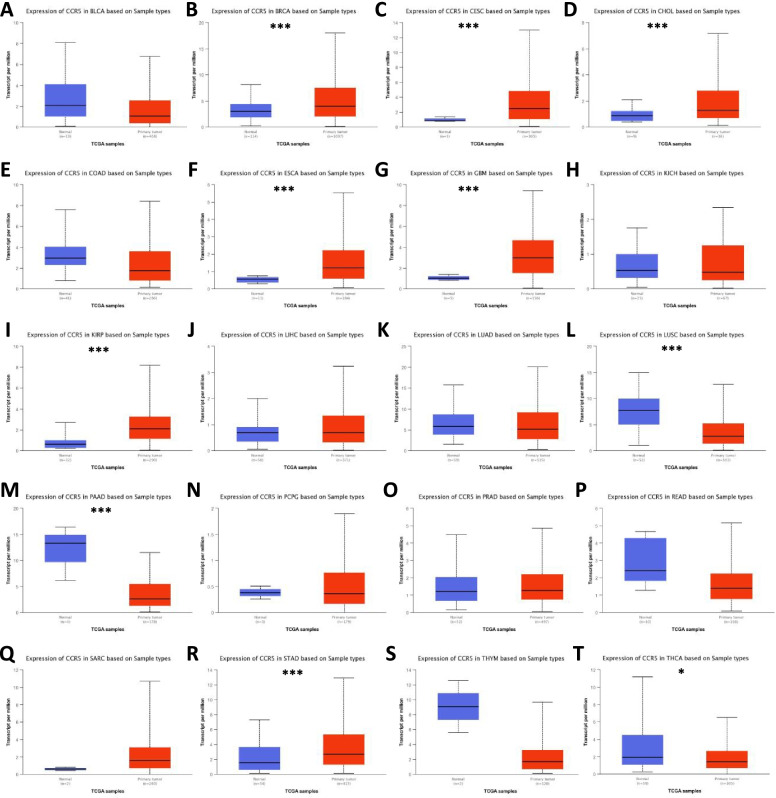
CCR5 mRNA expression levels in human various malignancies from TCGA database were determined by UCLAN (*p < 0.05, **p < 0.01, ***p < 0.001)

To investigate the expression levels of CCR5 in human various malignancies, we analyzed the RNA-seq data from various malignancies and the reciprocal normal specimens in TCGA using the TIMER database and the GTEx dataset. As shown in Fig. 1, we discovered that the expression levels of CCR5 were significantly upregulated in the tumor tissues of breast invasive carcinoma (BRCA), esophageal carcinoma (ESCA), glioblastoma (GBM), head and neck squamous cell carcinoma (HNSC), kidney renal clear cell carcinoma (KIRC), kidney renal papillary cell carcinoma (KIRP), pancreatic cancer (PAAD), stomach adenocarcinoma (STAD), lymphoid neoplasm diffuse large B-cell lymphoma (DLBC), skin cutaneous melanoma (SKCM), and testicular germ cell tumors (TGCT), while the expression levels were significantly downregulated in colon adenocarcinoma (COAD), lung squamous cell carcinoma (LUSC), and thyroid cancer (THCA). Fundamentally, the data of the UALCAN analysis indicated that the expression levels of CCR5 in various cancers was consistent with quantitative analysis of TCGA using TIMER database and the GTEx dataset (Fig. 2).

To further corroborate these results, tissue specimens from 520 HNSC tumors and 44 normal controls from the TCGA database were further analyzed using the GEPIA platform. As shown in Fig. 1, CCR5 expression levels were significantly increased in HNSC tissue specimens compared with normal control tissue specimens, which agrees with the results of the TIMER and UALCAN database. The TCGA database shows increased expression levels of CCR5 compared to matched normal tissues. Significantly, we found that the expression levels of CCR5 were consistent with PD1, and the AUC was 0.638 (95% CI: 0.571–0.705) for CCR5 in HNSC patients (Fig. 1). Taken together, our data demonstrated that CCR5 was significantly increased in HNSC patients and may be a potential diagnostic biomarker for HNSC.

### Correlation between upregulated CCR5 and clinical pathology in HNSC patients

**Fig. 3:**
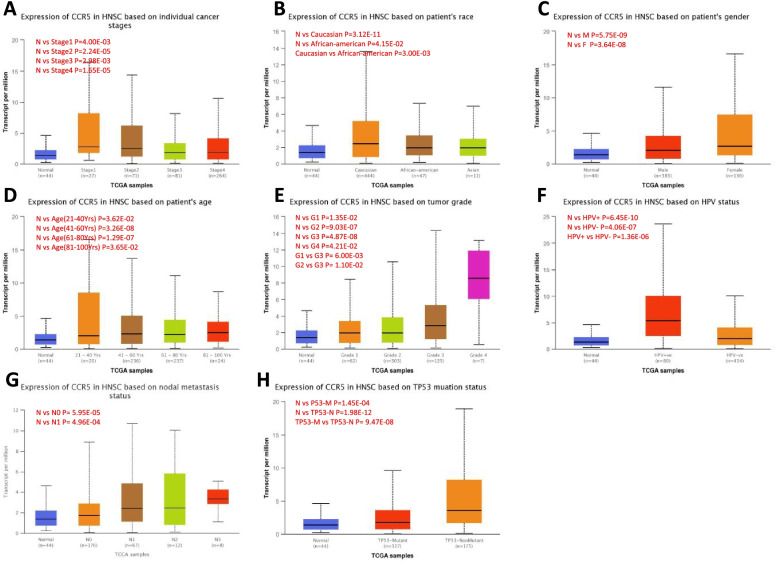
Correlation between CCR5 mRNA expression level and clinicopathological parameters of HNSC patients through the UALCAN database. **A** Cancer stage (stage 1, 2, 3, and 4). **B** Patient’s race (Caucasian, African-american, and Asian). **C** Patient’s gender (male, and female). **D** Patient’s age. **E** Tumor Grade (Grade 1, 2, 3, and 4). **F** Patient’s HPV status. **G** Nodal metastasis satus (N0 1, 2, and 3). **H** Patient’s TP53 mutation status

We further investigated the expression levels of CCR5 in several clinicopathological parameters using the TCGA HNSC dataset (Fig. 3). A study showed that the expression levels of CCR5 were significantly increased in the early-stage cancers than in the middle and late-stage cancers, indicating an underlying effect for CCR5 lies in the early detection of early-stage tumors. The expression levels of CCR5 in the patient’s race was significantly increased in Caucasians than in the other two races, suggesting that the expression levels of CCR5 is related to race. As a result, females had much higher CCR5 expression levels than males, and the expression levels of CCR5 in TP53 non-mutant types were also increased than that in TP53 mutant types. In particular, the expression levels of CCR5 were significantly increased at the lymph node stage than at all stages of tumor development. This suggests that CCR5 is existing in malignancies (Fig. 3).

Next, to further discover the underlying molecular mechanism of CCR5 in tumor development, the Kaplan-Meier plotter analysis was applied to dissect the relevance CCR5 expression levels with clinical prognosis (Table 1). In particular, we found that the significant upregulation of CCR5 was correlated with worse OS and RFS in male (OS, HR = 0.5, *p* = 2.4e-5; RFS, HR = 3.58, *p* = 0.0304). Furthermore, we observed that OS at stage 3 (HR = 0.32, *p* = 0.002) and stage 4 (HR = 0.67, *p* = 0.0268) correlated with CCR5 expression (Table 1). These findings manifested that the prognostic importance of CCR5 in HNSC patients depends on its clinical characteristics.

**Table 1:** Correlation of CCR5 mRNA expression and clinical prognosis in head and neck squamous cell carcinoma with different clinicopathological factors by Kaplan-Meier plotter

Clinicopathological characteristics	Overall survival (***n*** = 7462)			Recurrence free survival (***n*** = 4420)		
	N	Hazard ratio	***p***	N	Hazard ratio	***p***
**Gender**						
Female	133	0.65 (0.36–1.18)	0.153	46	2.03 (0.71–5.85)	0.1794
Male	366	0.5 (0.36–0.69)	**2.4e-5**	78	3.04 (1.06–8.75)	**0.0304**
**Stage**						
1	25	4.28 (0.44–41.33)	0.172	23	8.92 (1.54–51.79)	**0.0041**
2	69	0.65 (0.29–1.46)	0.2945	43	5.77 (1.06–31.58)	**0.022**
3	78	0.32 (0.15–0.68)	**0.002**	42	0.4 (0.11–1.46)	0.155
4	259	0.67 (0.47–0.96)	**0.0268**	/	/	/
**Race**						
white	426	0.62 (0.46–0.83)	**0.0012**	110	3.97 (1.76–8.89)	**0.0004**
asian	/	/	/	/	/	/
black/african america	47	0.51 (0.21–1.24)	0.131	/	/	/
**Grade**						
1	61	0.52 (0.19–1.44)	0.1991	26	6.65 (0.78–55.14)	**0.0471**
2	298	0.59 (0.42–0.84)	**0.0027**	69	2.37 (0.8–7.01)	0.1077
3	119	0.59 (0.33–1.07)	0.0776	25	5 (0.91–27.57)	**0.0408**
4	/	/	/	/	/	/
Mutation burden						
high	251	0.58 (0.4–0.83)	**0.0025**	50	5.58 (0.88–14.60	0.0592
low	234	0.57 (0.35–0.93)	**0.024**	73	3.05 (1.17–7.94)	**0.016**

*Note*. Bold values indicate *p* < 0.05

### Correlation between upregulated CCR5 and prognosis in HNSC patients

**Fig. 4:**
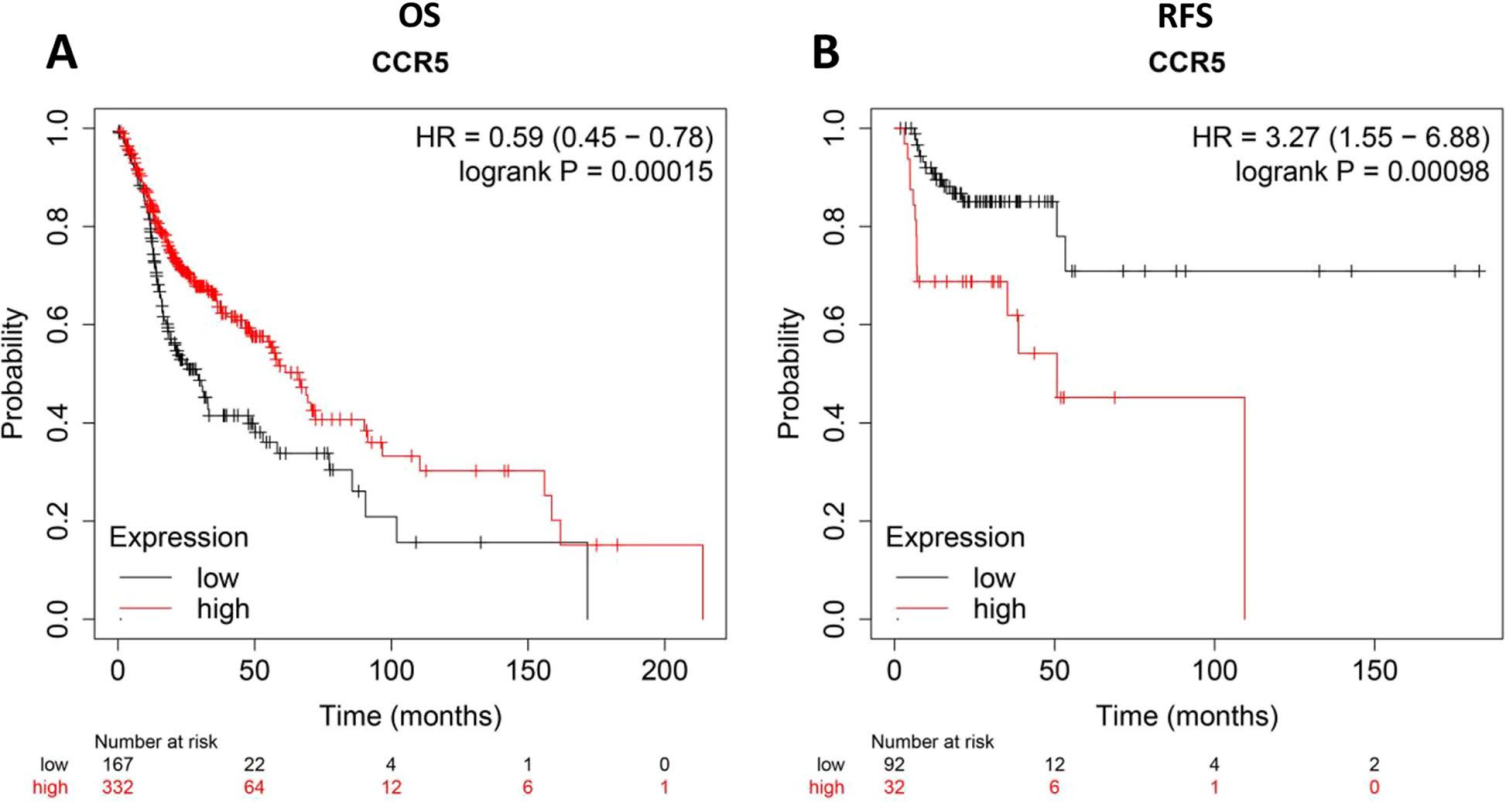
Kaplan-Meier survival curves comparing the high and low expression of CCR5 in HNSC in Kaplan-Meier plotter databases. (A). Survival curves of overall survival (OS, HR = 0.59, *p* = 0.00015) in the HNSC cohort. **B** Survival curves of recurrence-free survival (RFS, HR = 3.27, *p* = 0.00098) in the HNSC cohort

Then, the clinical prognostic significance of CCR5 in HNSC patients was determined using the KM plotter. The results of KM plotter analysis showed that the increased expression levels of CCR5 were dramatically correlated with a poorer prognosis in HNSC patients (OS, HR = 0.59, *p* = 0.00015; RFS, HR = 3.27, *p* = 0.00098), suggesting that CCR5 can serve as an underlying prognostic biomarker (Fig. 4).

### Correlation between upregulated CCR5 and immune infiltration in HNSC patients

**Fig. 5:**
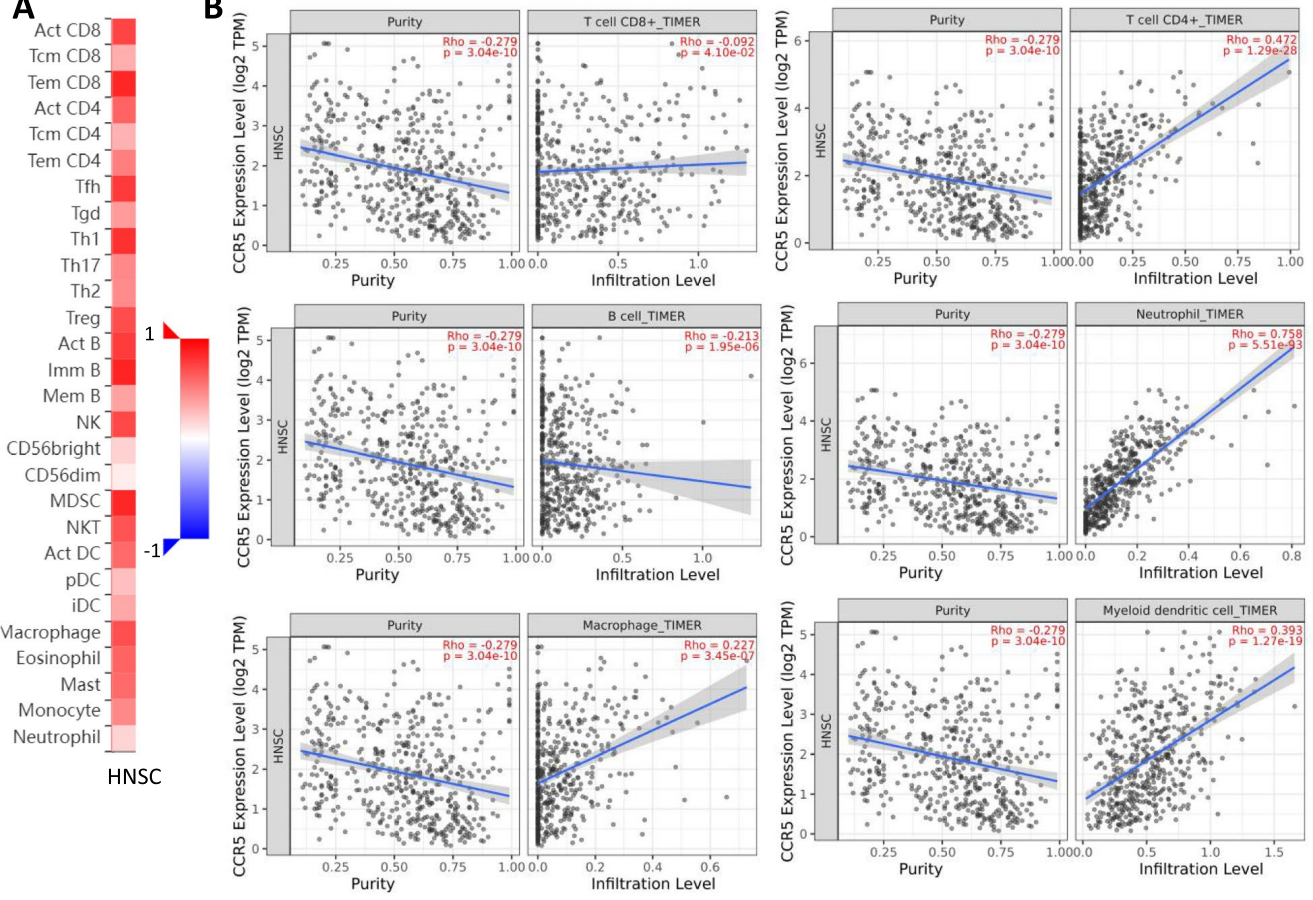
Correlation of CCR5 expression with immune infiltration in HNSC. **A** Correlation between the expression of CCR5 and the abundance of TILs in HNSC cancer available at TISIDB database. **B** Correlation of CCR5 expression with infiltration levels of CD8 + T cell, CD4 + T cell, B cell, neutrophils, macrophages, and myeloid dendritic cells in HNSC cancer available at TIMER2.0 database

Immune infiltration around tumors has been shown to be closely related to clinical outcomes in tumor patients. Therefore, we tested the relevance between the expression levels of CCR5 and immune infiltration in HNSC patients by TISIDB and TIMER platforms, determined whether the expression levels of CCR5 interrelated with immune infiltration level, and found that the upregulated CCR5 was significantly negatively interrelated with tumor purity (rho = − 0.279, *P* = 3.04e-10). Our data also found a strong relevance between the upregulated CCR5 and the abundance of TILs. For example, the upregulation of CCR5 in HNSC patients was positively associated with the infiltrating levels of CD4+ T cells (rho =0.472), neutrophils (rho = 0.758), macrophages (rho = 0.227), and the myeloid dendritic cells (rho = 0.393). Besides, the upregulated CCR5 was inversely correlated with CD8+ T cells (rho = − 0.092) and B cells (rho = − 0.213) infiltration levels (Fig. 5). As a result, our data indicated that CCR5 plays a significant function in the immune infiltration for HNSC patients.

Considering the important role of CCR5 in regulating immune infiltration in HNSC, we examined the relevance of the upregulated CCR5 with immunogenic biomarkers of TILs in HNSC patients using the TIMER and GEPIA databases. The data showed that the upregulated CCR5 positively correlated with the most immunogenic biomarkers of TILs in HNSC patients, particularly in CD8+ T cells (CD8A and CD8B), T cells (CD3D, CD3E, and CD2), B cells (CD19 and CD79A) and Monocytes (CD86 and CD115) (Table 2).

**Table 2:** Correlation analysis between CCR5 and related genes and markers of immune cells in Tumor Immune Estimation Resource (TIMER2.0)

Description	Gene markers	HNSC			
		None		Purity	
		Cor	***p***	Cor	***p***
CD8+ T cell	CD8A	0.896	9.19e-186***	0.887	1.58e-166***
	CD8B	0.846	5.64e-144***	0.832	2e-127***
T cell	CD3D	0.87	1.71e-161***	0.859	2.3e-144***
	CD3E	0.939	1.51e-242***	0.933	5.57e-220***
	CD2	0.938	4.26e-242***	0.932	1.49e-217***
B cell	CD19	0.588	6.64e-50***	0.558	1.52e-41***
	CD79A	0.582	1.26e-48***	0.553	8.95e-41***
Monocyte	CD86	0.772	2.16e-104***	0.749	1.37e-89***
	CD115 (CSF1R)	0.841	7.1e-141***	0.828	2.87e-125***
TAM	CCL2	0.587	1e-49***	0.553	1.01e-40***
	CD68	0.473	1.85e-30***	0.457	9.69e-27***
	IL10	0.618	2.29e-56***	0.58	1.28e-45***
M1 Macrophage	INOS (NOS2)	0.314	1.99e-13***	0.341	7.91e-15***
	IRF5	0.358	3.2e-17***	0.378	3.71e-18***
	COX2(PTGS2)	−0.147	7.7e-04***	−0.132	3.4e-03***
M2 Macrophage	CD163	0.71	3.84e-81***	0.701	8.05e-74***
	VSIG4	0.642	4.64e-62***	0.638	1.43e-57***
	MS4A4A	0.746	5.93e-94***	0.73	3.89e-83***
Neutrophils	CD66b (CEACAM8)	0.075	8.81e-02	0.059	1.9e-01
	CD11b (ITGAM)	0.633	9.83e-60***	0.614	2.08e-52***
	CCR7	0.749	3.41e-95***	0.726	1.37e-81***
Natural killer cell	KIR2DL1	0.398	2.86e-21***	0.4	2.39e-20***
	KIR2DL3	0.555	1.38e-43***	0.549	4.7e-40***
	KIR2DL4	0.639	2.37e-61***	0.639	7.05e-58***
	KIR3DL1	0.509	9.34e-36***	0.508	1.16e-33***
	KIR3DL2	0.651	2.58e-64***	0.644	5.71e-59***
	KIR3DL3	0.261	1.52e-09***	0.253	1.33e-08***
	KIR2DS4	0.409	1.85e-22***	0.386	6.8e-19***
Dendritic cell	HLA-DPB1	0.862	2.47e-155***	0.848	4.71e-137***
	HLA-DQB1	0.698	2.36e-77***	0.678	1.42e-67***
	HLA-DRA	0.858	1.69e-152***	0.844	2.04e-134***
	HLA-DPA1	0.864	7.54e-157***	0.851	5.61e-139***
	BDCA-1(CD1C)	0.54	7.3e-41***	0.489	6.57e-31***
	BDCA-4(NRP1)	0.431	4.4e-25***	0.415	6.67e-22***
	CD11c (ITGAX)	0.696	6.05e-77***	0.674	2.02e-66***
Th1	T -bet (TBX21)	0.896	5.71e-185***	0.888	2.99e-167***
	STAT4	0.681	1.74e-72***	0.652	7.99e-61***
	STAT1	0.623	1.97e-57***	0.605	1.95e-50***
	IFN-γ (IFNG)	0.759	4.45e-99***	0.743	2.28e-87***
	TNF-α (TNF)	0.227	1.62e-07***	0.2	7.51e-06***
Th2	GATA3	0.472	2.81e-30***	0.431	1.19e-23***
	STAT6	0.323	3.55e-14***	0.371	1.87e-17***
	STAT5A	0.661	9.69e-67***	0.654	2.84e-61***
	IL13	0.481	1.47e-31***	0.456	1.09e-26***
Tfh	BCL6	0.122	5.09e-03	0.185	3.61e-05***
	IL21	0.572	1.24e-46***	0.545	1.82e-39***
Th17	STAT3	0.395	6.09e-21***	0.399	2.85e-20***
	IL17A	0.362	1.23e-17***	0.337	1.5e-14***
Treg	FOXP3	0.853	9.12e-149***	0.839	1.05e-131***
	CCR8	0.752	4.31e-96***	0.728	1.89e-82***
	STAT5B	0.431	5.72e-25***	0.43	1.43e-23***
	TGFβ (TGFB1)	−0.012	7.89e-01	−0.028	5.42e-01
T cell exhaustion	PD-1 (PDCD1)	0.895	4.3e-184***	0.887	9.24e-167***
	PDL1(PDCD1LG2)	0.593	6.31e-51***	0.567	3.74e-43***
	CTLA4	0.823	1.06e-129***	0.808	1.07e-114***
	LAG3	0.817	2.22e-126***	0.807	2.88e-114***
	TIM-3 (HAVCR2)	0.879	2.91e-169***	0.873	6.53e-155***
	GZMB	0.776	3.73e-106***	0.759	2.31e-93***

*Note*. *HNSC* Head and neck squamous cell carcinoma, *TAM* Tumor-associated macrophage, *Th* T helper cell, *Tfh* Follicular helper T cell, *Treg* Regulatory T cell, *Cor* R value of Spearman’s correlation, *None* Correlation without adjustment, *Purity* Correlation adjusted by purity. *p < 0.01; **p < 0.001; ***p < 0.0001

**Fig. 6:**
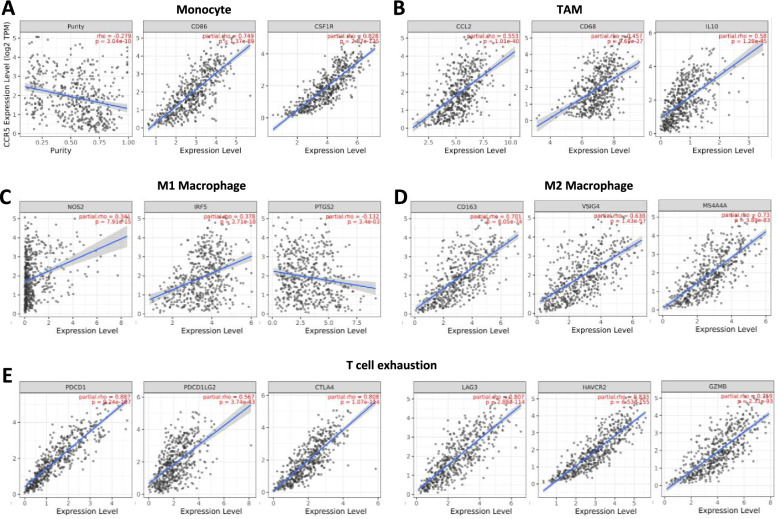
CCR5 expression correlated with monocyte, macrophages, and T cell exhaustion in HNSC patients. Immunogenic biomarkers include CD86 and CSF1R of monocytes; CCL2, CD68, and IL10 of TAMs; NOS2, IRF5, and PTGS2 of M1 macrophages; and CD163, VSIG4, and MS4A4A of M2 macrophages. Scatterplots of correlations between CCR5 expression and immunogenic biomarkers of monocytes (**A**), TAMs (**B**), and M1 (**C**), M2 macrophages (**D**) and T-cell exhaustion (**E**) in HNSC patients

The upregulated CCR5 was significantly associated with most immunogenic biomarkers of monocytes, TAMs, M1 macrophages, M2 macrophages, and T cell exhaustion in HNSC patients (Table 2). In particular, the results indicated that the expression levels of CCR5 was dramatically interrelated with monocytes (CD86 and CSF1), TAMs (CCL2, CD68, and IL10), M1 macrophages (NOS2 and IRF5), M2 macrophages (CD163, VSIG4, and MS4A4A), and T cell exhaustion (PDCD1, PDCD1LG2, CTLA4, LAG3, HAVCR2, and GZMB) (Fig. 6). In addition, based on the GEPIA database, we further investigated the relationship between the expression levels of CCR5 and immunogenic biomarkers such as monocytes, TAMs, M1 macrophages, M2 macrophages, and T cell exhaustion, which was similar to the TIMER platforms (Table 3).

**Table 3:** Correlation analysis between CCR5 and related genes and markers of monocyte, macrophages, and T-cell exhaustion in Gene Expression Profiling Interaction Analysis (GEPIA)

Description	Gene markers	HNSC			
		Tumor		Normal	
		Cor	***p***	Cor	***p***
Monocyte	CD86	0.77	4.7e-101^***^	0.67	7.6e-07^***^
	CD115 (CSF1R)	0.84	1.3e-136^***^	0.69	2.4e-07^***^
TAM	CCL2	0.58	4.9e-48^***^	0.12	0.45
	CD68	0.43	4.9e-25^***^	0.35	0.02
	IL10	0.63	2.8e-59^***^	0.56	7.9e-05^***^
M1 Macrophage	INOS (NOS2)	0.31	6.3e-13^***^	0.38	0.011
	IRF5	0.33	1.5e-14^***^	0.29	0.052
	COX2(PTGS2)	−0.14	0.0017^*^	0.08	0.6
M2 Macrophage	CD163	0.67	5.5e-70^***^	0.19	0.23
	VSIG4	0.63	3.9e-59^***^	0.2	0.18
	MS4A4A	0.74	1.7e-89^***^	0.22	0.15
T cell exhaustion	PD-1 (PDCD1)	0.9	7.1e-184^***^	0.76	2.2e-09^***^
	PDL1(PDCD1LG2)	0.59	7.6e-50^***^	0.51	0.00038^***^
	CTLA4	0.83	1.8e-133^***^	0.64	2.9e-06^***^
	LAG3	0.79	8.7e-113^***^	0.67	5.4e-07^***^
	TIM-3 (HAVCR2)	0.88	9.9e-167^***^	0.72	2.9e-08^***^
	GZMB	0.75	1.6e-94^***^	0.56	7.5e-05^***^

Note. *HNSC* Head and neck squamous cell carcinoma, *TAM* Tumor-associated macrophages; *Tumor* correlation analysis in tumor tissue of TCGA; *Normal* correlation analysis in normal tissue of TCGA. *p < 0.01; **p < 0.001; ***p < 0

### Correlation between upregulated CCR5 and immunomodulators in HNSC patients

**Fig. 7:**
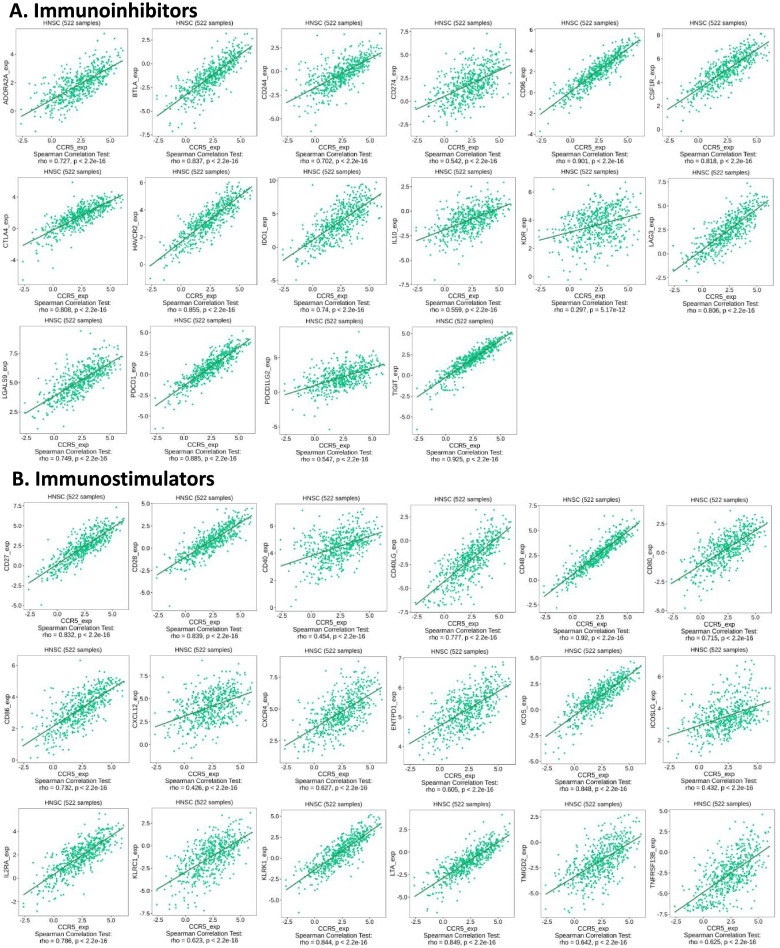
The expression of CCR5 is associated with immunomodulators in HNSC patients. **A** Correlation between CCR5 expression and immunoinhibitors in HNSC cancer available at TISIDB database. **B** Correlation between CCR5 expression and immunostimulators in HNSC available at TISIDB database

Significantly, we found that the upregulated CCR5 was positively correlated with immunoinhibitors in HNSC patients (*p* < 2.2e− 16), including ADORA2A (rho = 0.727), BTLA (rho = 0.837), CD244 (rho = 0.702), CD274 (rho = 0.542), and CD96 (rho = 0.901). The increased expression levels of CCR5 were also dramatically interrelated with immunostimulators in HNSC patients (*p* < 2.2e− 16), containing CD27 (rho = 0.832), CD28 (rho = 0.839), CD40 (rho = 0.454), CD40LG (rho = 0.777), and CD48 (rho = 0.92) (Fig. 7). These data shown that CCR5 is closely involved in the modulation of immune system interactions and may control tumor immune microenvironment (TME).

### Correlation between upregulated CCR5 and chemokines in HNSC patients

**Fig. 8:**
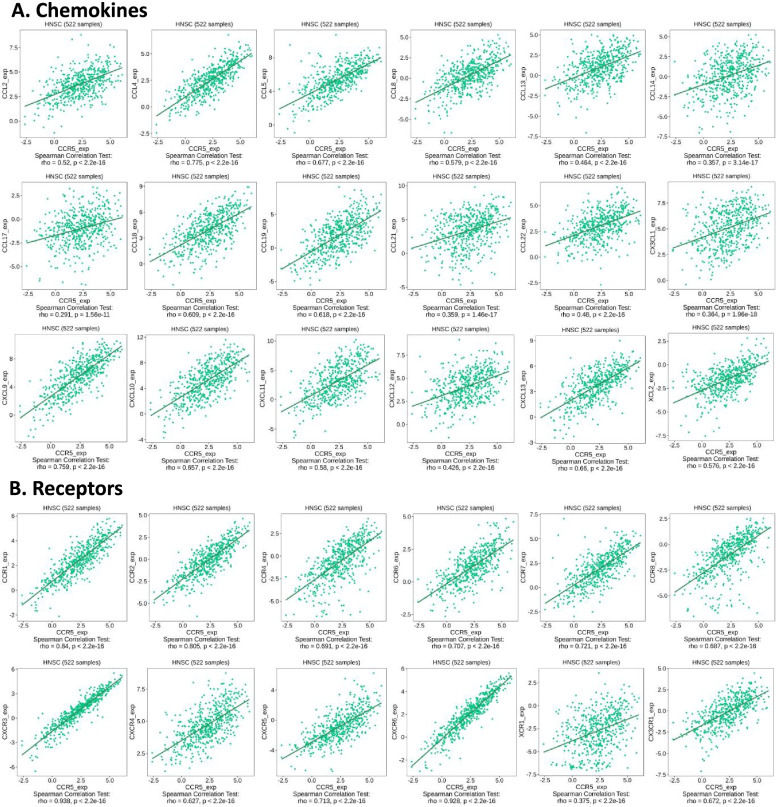
Correlation between the expression of CCR5 and chemokines in HNSC patients. **A** Correlation between CCR5 expression and chemokines in HNSC cancer available at TISIDB database. **B** Correlation between CCR5 expression and chemokine receptors in HNSC cancer available at TISIDB database

This study demonstrated the correlation of the expression levels of CCR5 with chemokines in HNSC patients. In particular, we found that the upregulated CCR5 was positively interrelated with chemokines in HNSC patients (*p* < 2.2e-16), containing CCL2 (rho = 0.52), CCL4 (rho = 0.775), CCL5 (rho = 0.677), CCL8 (rho = 0.579), and CCL13 (rho = 0.464). On the other hand, we found that the upregulated CCR5 was significantly interrelated with chemokine receptors in HNSC patients (*p* < 2.2e-16), containing CCR1 (rho = 0.84), CCR2 (rho = 0.805), CCR4 (rho = 0.691), CCR6 (rho = 0.707), and CCR7 (rho = 0.721) (Fig. 8). These findings further revealed that CCR5 may serve as an immunoregulatory element in HNSC patients.

## Discussion

HNSC is a very heterogeneous group of tumors with a poor prognosis. Despite major progresses in the diagnostic scheme and therapeutic approach of HNSC, prognosis remains difficult. It is increasingly clear that the TME plays a crucial function in HNSC tumorigenesis by promoting aggressive tumor development and therapy resistance [20]. However, in the absence of specific immune-related biomarkers, the five -year survival rates of HNSC patients remains low. In the current research, the clinical significance and the expression levels of CCR5 in HNSC patients were systematically analyzed by a global bioinformatics study. Our data suggested that the poor prognosis of HNSC is associated with the upregulation of CCR5 expression. Moreover, our results demonstrated that the upregulated CCR5 is closely related to the infiltrating levels of various immune cells, immunoinhibitors, immunostimulators, chemokines and receptors in HNSC. Therefore, our study provides a bran-new perspective on the basic effect of CCR5, and it may serve as a prognostic biomarker in HNSC patients.

As a seven-transmembrane G protein-coupled receptor, CCR5 can bind to a variety of ligands CCL3 (MIP1α), CCL3L1, CCL4 (MP-1β), CCL5 (RANTES), CCL8 (MCP2), CCL11 (Eotaxin), CCL13 (MCP-4), and CCL16, which are reported to be highly expressed in most malignant tumors [9]. With the new focus on tumorigenesis and persistent atypical signaling, CCR5 is currently being used as an emerging therapeutic target for most metastatic cancer, with current clinical experiments in breast and colon cancers [21, 22]. However, the underlying molecular mechanisms of action of CCR5 in HNSC tumors is unclear. Therefore, we detected CCR5 mRNA expression levels in various malignancies tissue specimens and normal control tissue specimens using the TIMER and GEPIA databases, and validated them with the UALCAN database. We discovered that CCR5 was expressed in both tumor tissue specimens and normal tissue specimens in several cancer types. The expression levels of CCR5 were significantly up-regulated in HNSC tissue specimens compared to paracancerous tissue specimens. The results are consistent with those of the TCGA database and other studies [23, 24]. We also found that the CCR5 mRNA levels were upregulated in most HNSC tissue specimens compared with matched paracancerous tissue specimens. Significantly, we found that the expression levels of CCR5 were consistent with PD1, and the AUC was 0.638 (95% CI: 0.571–0.705) for CCR5 in HNSC patients. In addition, to notarized whether CCR5 can serve as a prognostic biomarker, we explored the prognostic significance of CCR5 in HNSC patients using the Kaplan-Meier plotter analysis. The results indicated that the upregulated CCR5 was dramatically interrelated with a worse prognosis in HNSC patients, suggesting that CCR5 can serve as a potential prognostic biomarker for HNSC.

As immunotherapies primarily target the TME, we investigated the influence of CCR5 on immune cell infiltration of HNSC in the current research. Our results showed that the upregulated CCR5 in HNSC was positively interrelated with infiltrating levels of CD4+ T cells, neutrophils, macrophages, and myeloid dendritic cells. On the other hand, the upregulated CCR5 was associated with immunoinhibitors, immunostimulators, chemokines, and receptors. Furthermore, the present study also confirmed the correlation between the expression levels of CCR5 and the immunogenic markers of immune cells of HNSC. We clearly observed that the upregulated CCR5 was significantly positively interrelated with the immunogenic markers of monocytes (CD86 and CSF1), TAMs (CCL2, CD68, and IL10), M1 macrophages (NOS2 and IRF5), M2 macrophages (CD163, VSIG4, and MS4A4A), and T cell exhaustions (PDCD1, PDCD1LG2, CTLA4, LAG3, HAVCR2, and GZMB). Furthermore, the findings demonstrated that the upregulated CCR5 activates Treg and B cells, induces T cell exhaustion, promotes Treg responses, and suppresses T cell-mediated immunity, thereby modulating T cell responses in HNSC patients. The upregulated CCR5 can promote the polarization of macrophages towards M1 and M2 phenotypes. Overall, CCR5 plays a vital function in the recruitment and modulation of TILs in HNSC patients, and the underlying molecular mechanisms of action and function of CCR5 in regulating the TME deserve further study. In addition, further studies are still needed to investigate therapeutic targeting HNSC patients in the future.

## Conclusion

The upregulated CCR5 is strongly associated with worse prognosis and the infiltrating levels of various immune cells in HNSC patients. In addition, it helps to regulate the polarization of macrophages, and the exhaustion of T cells. Therefore, the present research demonstrates that CCR5 may serve as a prognostic biomarker, highlighting its underlying function in regulating TME in HNSC patients.

## Data Availability

All the datasets were retrieved from the publishing literature, so it was confirmed that all written informed consent was obtained. And all of other materials are available by the corresponding authors.

## Abbreviations

CCR5: C-C chemokine receptor 5
HNSC: Head and neck squamous cell carcinoma
TILs: Tumor-infiltrating lymphocytes
TIMER: Tumor Immune Estimation Resource
GEPIA: Gene Expression Profiling Interactive Analysis
TCGA: The Cancer Genome Atlas
OS: Overall survival
RFS: Recurrence-free survival
HPV: Human papillomavirus
R/M: Recurrent or metastatic squamous cell carcinoma
GPCR: G protein-coupled receptor
Tregs: Regulatory T cells
GTEx: Genotype-Tissue Expression
BRCA: Breast invasive carcinoma
ESCA: Esophageal carcinoma
GBM: Glioblastoma
KIRC: Kidney renal clear cell carcinoma
KIRP: Kidney renal papillary cell carcinoma
PAAD: Pancreatic cancer
STAD: Stomach adenocarcinoma
DLBC: Lymphoid neoplasm diffuse large B-cell lymphoma
SKCM: Skin cutaneous melanoma
TGCT: Testicular germ cell Tumors
COAD: Colon adenocarcinoma
LUSC: Lung squamous cell carcinoma
THCA: Thyroid cancer
TME: Tumor immune microenvironment
